# Biodemographic Analysis of Factors Related to Perinatal Mortality in Portugal (1988–2011)

**DOI:** 10.1155/2016/6123065

**Published:** 2016-10-27

**Authors:** Vicente Fuster

**Affiliations:** Complutense University of Madrid, Department of Zoology and Physical Anthropology, Faculty of Biology, Madrid, Spain

## Abstract

*Background*. The purpose of this paper is to determine the relative mortality risks at delivery and during the first week of life with regard to maternal and foetal characteristics.* Methods*. Yearly individual digital records on live births and early neonatal mortality were used to infer the possible factors involved in perinatal deaths.* Results*. The results show that the number of births per year declined with time throughout the period studied. At the same time, rates decreased in 66.4% for stillbirths and in 70.2% for early neonatal mortality. Logistic regressions modelled the interaction of the two mortality indicators and covariables such as birth weight and the duration of gestation.* Conclusions*. This research provides a first biodemographic approach to the knowledge of factors influencing perinatal mortality in Portugal based on a set of foetal and maternal variables. Although the magnitude of the different perinatal mortality rates may be affected by the criteria used for selecting cases (multiple-singletons; minimum birth weight or minimum duration of gestation), one of the conclusions of the present analysis is that the relationship among the maternal and foetal variables that determine the relative risk remains unaltered. Certain resemblance with the factors determining negative birth outcomes in Spain is appreciated.

## 1. Introduction

Perinatal mortality is estimated by the addition of stillbirths plus the early neonatal mortality, which represents deaths occurring during the first seven days after delivery. However, the criteria for determining both perinatal components are diverse. Due to the lack of uniformity in the birth outcome definitions, the comparison of rates among countries may be problematic [1]. This is true for stillbirths for which alternative definitions have been provided [2] depending on the application of thresholds for the gestational age or the birth weight, commonly 28 weeks or 500 g. The value of 28 weeks strictly represents late fetal deaths while early stillbirths are restricted to 20–27 weeks of gestation [3]. For a correct estimation of the stillbirth rates (stillbirths divided by stillbirths plus live births) the limits applied for considering the existence of a stillbirth should also be taken into account when determining the denominator, for example, including only stillbirths and live births of at least 28 weeks of gestation [4]. The advantage of calculating perinatal mortality rates by combining stillbirth with early neonatal mortality is the reduction of the bias due to the definition followed [5].

Although the World Health Organization (WHO) has tried to normalize the definition of perinatal mortality, discrepancies persist among countries [6]. According to some authors it is thought that gestational age is more decisive than birth weight concerning perinatal mortality, while taken into account a minimum birth weight could be affected by the average birth weight. For a set of European countries foetal mortality rates based on gestational ages of 28 weeks produced higher rates than those obtained considering birth weights of at least 1000 g. For the neonatal mortality rate the influence of the criteria applied is less relevant [1]. In a different study, cases with birth weight < 500 or < 1000 g and gestational ages < 24 or < 28 weeks were analyzed [7], while in another [8], it was concluded that because it is a predictor of maturity and viability, a minimum gestational age threshold such as 28 weeks would be more appropriate than a birth weight criterion.

For national statistics the inscription of stillbirths and live births could vary and low thresholds such as gestation ages of at least 22 weeks or birth weights ≥500 g are acceptable. However, for intercountry comparisons standardization should be preferable in agreement with WHO recommendations: ≥28 weeks or ≥1000 g [6].

With regard to the factors possibly determining perinatal mortality, weight and prematurity are recognised as the most important conditions causing stillbirths [9]. The likelihood of stillbirth starts to accelerate as mothers reach the age of 35 and increases with age, raising more quickly after age of 40 [10].

Socioeconomic and cultural factors are also reported to have an influence on birth outcomes. Stillbirth rates tend to increase in association with drug abuse [11], which is more prevalent among less educated women [12].

Unfavourable working conditions result in low socioeconomic status which in turn leads to low levels of schooling. Both factors induce an increased risk of stillbirth [13]. Workers with lower social status in Sweden had a higher risk of stillbirth compared to senior white-collar workers [14]. This issue has also been studied in terms of maternal origin and ethnic group [15, 16]. Working conditions among immigrants can be expected to be less favourable than those of the native population.

In Portugal, as in other developed countries, there has been a significant delay in reproduction in recent decades [17] relating to the second demographic transition and caused by women's greater interest in pursuing a professional career [18], which resulted in low fertility. Thus, in 2012 the Directorate General of Health (Portugal) reported a general birth rate of 36.29 per 1000 women of childbearing age (15–49 years). Delayed childbearing has increased the proportion of births in primiparous women over 35, and the frequency of multiple deliveries due to the use of assisted reproductive technology [19]. Perinatal outcomes of pregnancies occurring after assisted reproduction techniques are known to be less successful than those following natural conception [20].

One of the first publications on Portuguese newborn characteristics was by Padez and Rocha [21] and consisted of 1232 women delivering at a small village maternity unit. Low weight at birth has also been studied using information from maternity wards to prove its adverse influence on certain diseases [22–26]. Neonatal mortality in very low birth weight deliveries has benefited from the implementation of a regionalisation policy in Portugal, which has led to the positive evolution of perinatal and neonatal mortality [27]. Descriptive studies have been published based on hospital records [3, 28–30]. The concordance among observations in public maternity wards and national records was assessed by Alves et al. [31]. A progressive downward trend in mean birth weight in Portugal in recent years was reported [32], which was attributed to shifts in the duration of gestation. Portuguese mothers over 35 years were also associated with a higher incidence of low birth weight, and with delivering a newborn of parity 1 (without previous children).

Although in Western Europe perinatal mortality has benefited by the improvement of medical care of pregnancies and proper attendance during deliveries, conducting to low mortality rates, survival at birth is expected to continue being highly influenced by sociodemographic, reproductive, and cultural factors. The present study is not addressed to quantify perinatal mortality rates (stillbirths and early neonatal). This information is available in several articles based on data in last term provided by the National Institute for Statistics of Portugal, for instance, those of Graafmans et al. [6] and Zeitlin et al. [33]. These papers provide comparable mortality rates after selecting gestations ≥28 weeks for determining stillbirth rates. The same occurs with Robalo et al. [3] report based on data from a tertiary-perinatal referral maternity. Accordingly, our paper aims to provide a first biodemographic analysis of the perinatal mortality in Portugal regarding their possible determinants in the general population, such as maternal age at delivery, child's birth weight, and prematurity. In addition, the existence of an Iberian Peninsula pattern (Portugal plus Spain) is to be checked. Analyzing individual data on pregnancies instead of aggregated data constitutes a different approach which intends to complement the information coming from a medical perspective.

## 2. Materials and Methods

The information here analyzed was supplied by the Portuguese Institute of Statistics (INE). Data were not aggregated as in most overall population analysis; instead they consisted of individual digital records for each live birth and perinatal death (microdata), appearing as yearly files from 1988 to 2011. After combining births and deaths registers, a data base of 2,618,467 records was obtained (one record per pregnancy).

In Portugal the National Institute for Statistics registers foetal mortality for gestational ages of 22 weeks and more. However, in the microdata records variations were detected in the format of the yearly perinatal mortality files and in the codification of variables. Depending on the characteristics of the data the initial yearly files were grouped into three sets: 1988–1995, 1996–2009, and 2010-11. The quality of the records between 1996 and 2009 was poor (many missing cases) which made it impossible to consider certain variables such as the duration of gestation for these years.

For weight at birth the following categories were considered: 500–1499 g; 1500–2499 g; 2500–4000 g; >4000 g. For particular analysis values less than 500 grams were discarded; that was the case for a live birth of 300 grams or a new born at term of 400 g. Discarded cases are very scarce; for instance, in 2011 they were 0.0124%.

**Figure 1:**
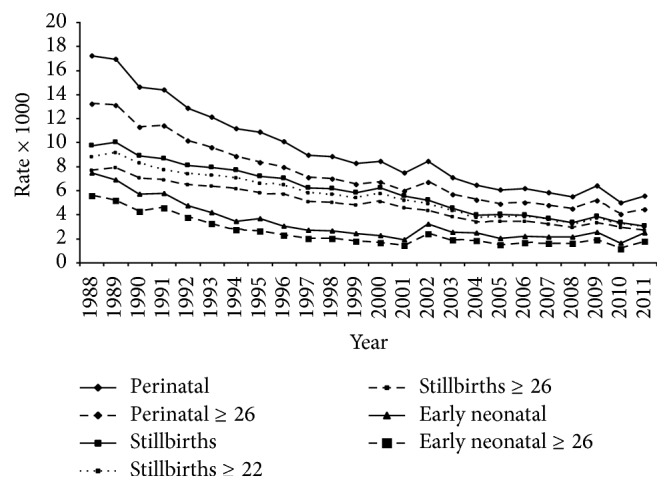
Yearly stillbirth and early neonatal and perinatal mortality rates (×1000) for total deliveries and gestations ≥22 and ≥26 weeks. Period 1996–2009: values of gestations ≥26 weeks estimated by linear regression on the total rates (corrected* R* ^2^ = 0.998, 0.986 and 0.992, resp., for each type of mortality). For stillbirths, also gestations ≥22 weeks estimated by linear regression on the total rate (corrected* R* ^2^ = 0.998).

The duration of gestation in the microdata files did not appear always as quantitative variables (weeks) but categorical (merged into categories). For the years 1988–1995 and 2010-2011, some few cases in which the duration of gestation was lower than 22 weeks were discarded, and the following groups were considered: 22–31; 32–36; 37–40 and >41 weeks. For the years where the duration of gestation was unknown, mortality rates for pregnancies lasting 22 or 26 weeks or more were estimated by linear regression in order to obtain the values represented in Figure 1.

Parity (birth order) is as follows: parity = 1 in case of a first birth (nulliparous); parity = 2 for a second or subsequent births (multiparous).

Maternal age is as follows: <20; 20–29; 30–39; 40–49 years. 179 mothers aged 50 or older were discarded as no stillbirths were found for this age group.

Once records with missing values of the variables to be analyzed were excluded, the original database reduced to 2,547,366 valid cases.

Forward (Wald) binary logistic regressions were applied, taking stillbirths and early neonatal and perinatal deaths separately as the dependent variables. Stillbirth was defined as a baby born with no signs of live while early neonatal mortality represents deaths occurring in the first seven days after delivery. Perinatal mortality is given by the sum of both types of deaths. Although the WHO recommends a threshold of 28 weeks' gestation in order to obtain international comparable values, in the present study particular criteria of selection were applied for considering they could better reflex the interaction of maternal-foetal variables as well as discarding wrong values.

For covariables, the reference category was assigned to the group with the lowest mortality rate: type of delivery (single), sex (females), duration of gestation (37–41 weeks), birth weight (2500–4000 grams), maternity age (<20 years), and parity (2 or more). The year was taken as a quantitative variable, and the reference was the first year analysed (1988).

Due to the incompleteness of the records, two types of regressions were performed:

- For the period of 1988–2011, the duration of gestation and parity could not be used as covariables.
- For years 1988–1995 and 2010-2011, duration of gestation (≥22 weeks) and parity (nulliparous-multiparous) were added as covariables.

## 3. Results 

For the period 1988–2011, the number of total births (live and stillbirths) estimated from the microdata files was equal to 2,547,366. In Table 1 are indicated the annual numbers of stillbirths and early neonatal deaths as well as the corresponding rates per 1000 births (only live births in case of neonatal). The overall frequencies of stillbirths and early neonatal deaths were 15,944 (6.26 per 1000) and 8,256 (3.26 per 1000), respectively. The yearly number of births declined from 118,711 in 1988 to 90,557 in 2011. Throughout time rates decreased in 66.4% for stillbirths and in 70.2% for early neonatal mortality. The proportion of stillbirths to perinatal mortality was high up to 2001 where the proportion was 3.2 times larger. Since then, the proportion decreased to the half.

Stillbirths deserve particular attention due to their notable contribution to perinatal mortality. Thus in order to obtain rates comparable to those available for the country closest to Portugal (Spain), where compulsory inscription covers only those who are stillborn of 26 or more weeks [34], the rates were recalculated including only pregnancies of 22 or 26 weeks' gestation. Figure 1 shows the rates corresponding to overall perinatal and early neonatal deaths (less than 7 days after delivery) and stillbirths regardless of the duration of gestation (crude rates) and for ≥26 weeks (also ≥22 weeks for stillbirths).

The annual crude stillbirth rates and the estimated rates for gestations of at least 22 or 26 weeks shown in Figure 1 indicate that the proportion of pregnancies of 26 weeks or more is 76.3% of the total stillbirth rate in 1995 and 96.92% in 2011. This figure reveals a change in trend in 2004, after which rates remained somewhat stable, at around 3 per 1000.

Table 2 shows the stillbirth rates for various categories of variables. Aside from the duration of gestation, the risk of stillbirth depends on the child's sex. For merged data, stillbirth rates in Portugal are unfavourable for males.

Births from multiple pregnancies are observed to be associated with more frequent stillbirths. All these groups had lower rates at the end of the period analysed, particularly in the case of multiple deliveries.

In the present study, a higher stillbirth risk was found for women who had no previous deliveries (nulliparous) than in women who had experienced maternity. The same applies for late maternities.

A priori, the duration of gestation reveals the greatest influence on that rate, followed by weight at birth. Other variables included in this table such as maternal age, parity, marital status, being born from a multiple gestation, and sex appear to play a less important role.

A set of binary logistic regressions were computed in order to model the interaction of the above variables, using the existence of a stillbirth as the dependent variable (Tables 3(a) and 3(b)).

Since some studies exclude multiple deliveries from the analysis while others do not, these tables show the regression parameters with and without multiple deliveries. Due to the deficiencies in the data, this first regression does not take into account the child's degree of maturation. The “Wald” column in the table corresponds to a statistic based on the chi square test, in which high values indicate a strong relationship between a covariable and the dependent variable. The proportion of variation explained by the model (from 0 to 1) is given by the Negelkerke* R* ^2^ coefficient. According to this regression, very low birth weight represents the highest risk factor, followed at a distance by low birth weight (1500–2499 grams). The relative risk obtained from these two models is mostly unaffected by the inclusion or exclusion of multiple deliveries as a covariable. For the years 1988–95 and 2010-11, duration of gestations ≥ 22 and parity were included in the models (Table 3(b)), again considering or excluding multiple deliveries. Although the percentage of variability explained by the models (Nagelkerke* R* ^2^) is greater in the case of gestations ≥ 22 weeks, the inclusion of these two variables, mostly parity, does not substantially alter the relative importance of risks attributed to each category of variables: very low birth weight remains the highest, followed by low birth weight and very premature pregnancies. The exclusion of multiple deliveries very slightly reduces* R* ^2^. The results in Table 3(b) reveal that in Portugal low weight and prematurity are the most important risk factors for stillbirth.

Early neonatal mortality denotes deaths occurring before the seventh day after delivery and, in combination with stillbirths, constitutes perinatal mortality. The distribution of the number of hours of survival in Table 4 shows that about 50% of deaths occurred during the first day of life and that no clear temporal pattern of change can be observed, although cases of survival for 72 hours increased slightly in 2010-11. The lesser contribution of early neonatal mortality to perinatal mortality over time is therefore due to an early decrease in this category of deaths, rather than to a variation in survival duration.

Tables 5(a) and 5(b) show the results of the logistic regressions taking early neonatal and perinatal deaths as the dependent variable and using as factors the same set of variables and categories as for stillbirths. The values in this table fairly closely resemble the rates for stillbirths. Very low birth weight, low birth weight, and very brief pregnancies continue to be the main risk factors. Multiple deliveries show OR > 1 in contrast to stillbirths. The same considerations apply to perinatal deaths, that is, stillbirths plus early neonatal deaths.

## 4. Discussion

The frequency of stillbirths and overall early neonatal deaths decreased from 1888 to 2011 when stillbirth rates were equal to 3.52. The rates obtained from microdata (9.70 in 1988) may be compared with those reported for 1994 [6], using values for pregnancies at least 28 weeks published by the Portuguese National Institute for Statists (1996), 9.2 (8.6–9.8), as well as Eurostat Demographic Statistics (1997), 9.3 (8.7–9.8).

For pregnancies at least 28 weeks stillbirths rates of 2.69 (2.4–3.0) were reported for the year 2004 [1]. This rate was very close to that obtained after applying a birth weight criteria (at least 1000 g): 2.64 (2.3–2.9). In Portugal, as in other developed countries complications during pregnancy are few and outcomes are generally favourable for both mothers and infants [35]; stillbirths currently account for more than 50% of all perinatal deaths [36].

Although it is difficult to set a range of limit continuous variable such as foetal maturation [37], the relative contribution of stillbirths to perinatal mortality increased throughout time up to 2001 where the proportion was 3.2 times larger. After that, the proportion decreased to values over 1.5 (Table 1). Better prenatal medical following of pregnancies could explain the temporal tendency observed in the relationship between death at delivery and those along the first week of life. But also increased survival to birth may have been taken to delayed mortality occurring during the first week of life. In fact 50% of early neonatal death occurred during the first 24 hours after delivery (Table 4).

International differences in countries' published perinatal mortality rates partly reflect the differences between each country's criteria for registering perinatal deaths [6]. To check the existence of an Iberian perinatal mortality pattern, Figure 1 represents rates for Portugal and Spain, where compulsory inscription covers only those who are stillborn of 26 or more weeks [34]. The temporal reduction in perinatal mortality is evident irrespective of duration of gestation. However, when valid cases are restricted to longer maturation ages, the curves move nearer the* x*-axis. The rates shown in Figure 1 indicate a change in trend in 2004, after which rates remained somewhat stable, at around 3 per 1000. In singleton pregnancies of 26 weeks or more, Fuster et al. [34] reported stillbirth rates of 2.7 (×1000) for the years 2007–2012 in Spain, slightly lower than for Portugal (2010-2011), but in this case all type of pregnancies were considered.

Birth weight in Portugal fell between 1995 and 2011, mainly due to the higher proportion of preterm births [32]. At the same time, the proportion of multiple deliveries increased. The same phenomenon was observed for birth weight since 1980 in Spain [38]. Although the greater frequency of low birth weights and multiple deliveries in Portugal could be considered unfavourable factors for birth outcomes, the reduction in perinatal mortality can be attributed to a more efficient prevention of prenatal and neonatal mortality, leading to a greater proportion of live premature deliveries [39].

Table 2 showed 1988 to 2011 merged data that state that stillbirth rates in Portugal are more frequent for males. However, yearly rates were not systematically more elevated for males. This only happened in 14 of the 24 years studied. It has been suggested that the males' disadvantage may be related to side effects stemming from their greater average size. Slight differences were detected in male/female who were stillborn for any factor related to survival, such as mother's age, education, birth order, and length of gestation [40].

The more elevated stillbirth risk found for nulliparous mothers may be attributed to obstetric complications which are more frequent in first deliveries when there is a higher risk of delivering low birth weight infants and infants with restricted intrauterine growth [41]. Also, higher risk associated with late maternity could be related to a greater demand for reproductive treatments, which significantly increase the risk of stillbirths [42].

Tables 3(a) and 3(b) show that besides sex and multiple births other factors such as duration of gestation and weight at birth can be expected to relate to the risk of stillbirth. A priori, the duration of gestation reveals the greatest influence on that rate, followed by weight at birth. Other variables included in this table such as maternal age, parity, marital status, being born from a multiple gestation, and sex appear to play a less important role.

The binary logistic regressions appearing in Table 3(a) consider or ignore multiple deliveries but the child's degree of maturation was not taken into account in this table because of lack of information. The relative risk obtained from these two models is mostly unaffected by the inclusion or exclusion of multiple deliveries as a covariable. Luke and Brown [43] reported that stillbirths have a complex multifactorial condition and are influenced by parity, weight at birth, and the duration of gestation, in addition to maternal age. For the years 1988–95 and 2010-11 the percentage of variability explained by the models (Nagelkerke* R* ^2^) is greater in the case of gestations ≥ 22 weeks; the inclusion of these two variables, mostly parity, does not substantially alter the relative importance of risks attributed to each category of variables: very low birth weight remains the highest, followed by low birth weight and very premature pregnancies. The exclusion of multiple deliveries very slightly reduces* R* ^2^. The results in Table 3(b) reveal that in Portugal low weight and prematurity are the most important risk factors for stillbirth, which agrees with the findings of Mohsin et al. [9].

With regard to maternal age, a study in Italy found that adverse foetal outcomes start to accelerate after mothers reach the age of 35 and increase with age, rising more quickly from the age of 40 [10]. This is also true for Portugal, as shown in Tables 3(a) and 3(b). However, this is not as evident for mothers aged over 40 when considering relative risks from the logistic regression.

Early neonatal mortality denotes deaths occurring before the seventh day after delivery and, in combination with stillbirths, constitutes perinatal mortality. The distribution of deaths during the first seven days after birth resulted in an accumulation of cases during the first day of life (Table 4). The lesser contribution of early neonatal mortality to perinatal mortality over time is therefore due to an early decrease in this category of deaths, rather than to a variation in survival duration.

The results obtained from the logistic regression analyses indicate close patterns for stillbirths (Table 5(a)) as well as for the perinatal mortality (Table 5(b)). In both cases the set of variables included in the model to represent the main risk factors were the same: very low birth weight, low birth weight, and very brief pregnancies continue to be the principal risk factors. A difference was observed with regard to multiple deliveries which associate with increased risk in case on early neonatal mortality. Analyzing stillbirths and early neonatal mortality together gave the same results as those obtained when analyzing each of them separately.

Late childbearing in Portugal, as in most developed countries, has become predominant. This affected mostly women with an advanced education, who frequently chose voluntary postponing of pregnancies for personal or professional reasons [17]. Late childbearing also may be related to a greater demand for reproductive treatments which result in an increased stillbirth risk [42].

Concerning parity, a greater stillbirth risk was found for nulliparous women than for those who had previously had children. Obstetric complications are more frequent at first delivery for which there is a higher risk of low birth weight infants and intrauterine-growth-restriction [41].

In comparison with Spain, several papers have intended to find similitudes between the Portuguese and Spanish biodemographic patterns respecting multiple deliveries and birth weight [32, 38]. The yearly evolution of double and triple deliveries in Portugal and its territorial distribution resemble that of Spain [32, 38]. In addition, the factors determining multiple births are similar in both countries, indicating an Iberian Peninsula pattern. Differences found in recent years are consistent with a more extensive use of assisted reproductive technology cycles [44].

A higher incidence of low birth weight occurred among Portuguese mothers over 35 years. Moreover, being a newborn of parity 1, and with the mother not in a couple, resulted frequently in more low birth weight. From 1988 to 2011 there was in Portugal a progressive reduction in the average weight at birth related to changes in the duration of gestation. An initial decline in the frequency of postterm births took place, followed by small variations from 1995 on. Long gestation periods and having reached a secondary or university education constituted a favorable factor regarding birth weight [32].

With regard to stillbirths and the socioeconomic conditions, it was reported for Spain that those mothers residing in regions with high unemployment had a greater chance of stillbirth [15]. It was also found that the mother's level of schooling was an indicator of unfavorable socioeconomic conditions. The relationship between schooling and stillbirths varies depending on maternal age. Spanish women with limited schooling (less than 5 years) show a higher risk of stillbirth than the non-Spanish. This finding is possibly related to the fact that in Spain women with less than 5 years of schooling come from very low socioeconomic segments of the population [34]. Contrarily to the studies on Spain, socioeconomic covaribles such as the marital status and the mothers' education were not included in the models for Portugal. Moreover, multiple pregnancies did not appear as determinant for stillbirths. However, early neonatal mortality showed a certain influence. Very low birth weight and maternal ages older than 30 and short gestations (for the years with available data) appeared as significant factors explaining the risk both of stillbirth or neonatal mortality.

## 5. Conclusion

Regardless of the model applied in this analysis, a relative risk (R.R) < 1 was always found for the year. This agrees with the temporal variation shown in Figure 1 and consists of a rapid initial decrease followed by a steady variation in subsequent years. This recalls the results reported by Gregory et al. [45] in the United States, where after declining from 2000–2006 overall (≥20 weeks), early (20–27 weeks), and late (≥28 weeks) foetal mortality rates remained essentially unchanged for 2006–2012. Although the situation in the labour market may have negatively affected the unemployed population [46], improvements in medical neonatology should have been sufficient to maintain low levels of perinatal mortality.

In a Portuguese retrospective analysis of 208 singleton stillbirths delivered in a tertiary-perinatal referral maternity unit over a 10-year period (2000–2009), Robalo et al. [3] found that the primary cause of death was foetal, and there was no temporal change in the incidence of late stillbirth. The change described here for Portugal is explained by the fact that the country's perinatal care was reformed in 1989 in order to coordinate local health centres and hospitals [47]. A national system was set up for neonatal transport to hospital maternity units (levels II and III), in addition to postgraduate courses on neonatology. After this reform, hospital deliveries became practically universal, and perinatal mortality decreased substantially. This is consistent with the situation observed in most western European countries as shown by the EUROSTAT databases [48], where in 2011 Portugal shows late foetal and early neonatal mortality rates below the European Union (28 countries) average and close to the values for Spain, Italy, and Greece. Although at present in Portugal the perinatal mortality is very low, multiple logistic regressions show that low birth weight (<1500 g) and maternal ages older than 30 and gestations <31 weeks remain the main factors determining the risk of a stillbirth or a neonatal death. The application of different criteria for selecting cases (multiple-singletons; minimum birth weight or minimum duration of gestation) does not modify significantly the above models. Certain similarities with the factors determining negative birth outcomes in Spain are appreciated. Regarding population preventive politics particular attention should be addressed to mothers older than 30 delivering children of low or very low birth weight or premature or extreme premature. No risk associated to nulliparity was found in this study.

## Figures and Tables

**Table 1:** Yearly frequency of stillbirths, early neonatal deaths, and total births. Rates of stillbirths and early neonatal deaths. Proportion stillbirth rate/early neonatal rate.

Year	Stillbirths	Stillbirths rate	Early neonatal	Early neonatal rate	Total births	Proportion
1988	1152	9.70	816	6.94	118711	1.40
1989	1156	10.04	724	6.35	115110	1.58
1990	1010	8.94	598	5.34	112931	1.67
1991	984	8.72	616	5.50	112897	1.58
1992	909	8.15	494	4.46	111548	1.83
1993	887	8.04	419	3.83	110345	2.10
1994	825	7.81	327	3.12	105577	2.50
1995	747	7.22	333	3.24	103490	2.23
1996	759	7.13	323	3.06	106424	2.33
1997	692	6.35	299	2.76	108937	2.30
1998	687	6.28	288	2.65	109339	2.37
1999	671	6.03	263	2.38	111270	2.54
2000	696	6.05	255	2.23	115135	2.71
2001	662	6.14	206	1.92	107809	3.19
2002	592	5.41	342	3.14	109446	1.72
2003	508	4.74	264	2.48	107159	1.92
2004	428	4.13	259	2.51	103667	1.65
2005	434	4.18	215	2.08	103781	2.01
2007	377	4.18	212	2.24	97027	1.86
2008	341	3.89	217	2.19	97913	1.77
2009	381	3.48	243	2.22	93573	1.57
2010	334	4.07	133	2.61	94936	1.56
2011	295	3.52	187	1.41	90557	2.50

Stillbirths and early neonatal deaths regardless of duration of gestation. Early neonatal deaths: under one week of life. Denominator for the stillbirth rate is equal to the sum of live births plus stillbirths. Denominator for the early neonatal mortality rate is equal to live births.

**Table 2:** Stillbirth rates ×1000 (S.B.) by category group for each variable studied (1988–2011).

Variable	Group	Total	S.B. rate
Duration of gestation (weeks)	22–31	4887	593.820
	32–36	15058	137.336
	37–41	184945	13.691
	>41	838	194.511

Maternal age (years)	<20	173046	4.432
	20–29	1426046	4.889
	30–39	1013607	6.564
	40–49	62862	15.287

Birth weight (grams)	<1500	27625	227.982
	1500–1249	163634	24.341
	2500–4000	2323280	1.867
	>4000	151848	2.127

Sex	Male	1379784	6.095
	Female	1297595	5.724

Type of delivery	Single	2615518	5.711
	Multiple	61968	16.266

Parity	1st	1410194	3.182
	2nd or more	1259135	2.981

Marital status	In couple	1161861	3.903
	Not in couple	477666	6.048

Duration of gestation: 1996–2009 is not considered. Marital status: values since 1997.

**(a):** 

Covariable	df	With multiple			Only single		
		Wald	*p*	OR	Wald	*p*	OR
Multiple (S)	1	51	*∗∗*	0.755	—	—	—
Sex (female)	1	43	*∗∗*	1.126	41	*∗∗*	1.127
BW (2500–4000)	3	58051	*∗∗*		57026	*∗∗*	
>4000	1	206	*∗∗*	1.933	189	*∗∗*	1.897
1500–2499	1	15189	*∗∗*	16.626	14839	*∗∗*	16.857
<1500	1	56348	*∗∗*	212.788	55134	*∗∗*	217.684
Mat. age (<20)	3	2949	*∗∗*		2804	*∗∗*	
20–29	1	92	*∗∗*	1.482	86	*∗∗*	1.472
30–39	1	993	*∗∗*	3.711	966	*∗∗*	3.734
40–49	1	8	*∗∗*	1.155	12	*∗∗*	1.206
Year (1988)	1	3223	*∗∗*	0.922	2884	*∗∗*	0.925
*Nagelkerke R* ^2^		0.312			0.309		

In parentheses the reference category (S: single; BW: birth weight; weeks: duration of gestation). df = degrees of freedom; *p* = statistical significance *∗∗* < 0.01. OR = relative risk. Wald values taken to the nearest integer. Nagelkerke corrected *R* ^2^ for total and single deliveries.

**(b):** 

Covariable	df	With multiple			Only single		
		Wald	*p*	OR	Wald	*p*	OR
Multiple (S)	1	4	*∗*	0.891	—	—	—
Sex (female)	1	4	*∗*	1.055	5	*∗*	1.058
BW (2500–4000)	3	6715	*∗∗*		6379	*∗∗*	
>4000	1	208	*∗∗*	2.272	190	*∗∗*	2.227
1500–2499	1	4091	*∗∗*	9.493	3972	*∗∗*	9.576
<1500	1	5880	*∗∗*	45.880	5463	*∗∗*	44.503
Mat. age (<20)	3	584	*∗∗*		538	*∗∗*	
20–29	1	11	*∗∗*	1.171	12	*∗∗*	1.177
30–39	1	197	*∗∗*	2.058	188	*∗∗*	2.058
40–49	1	28	*∗∗*	0.679	25	*∗∗*	0.686
Weeks (37–41)	3	2102	*∗∗*		2130	*∗∗*	
>41	1	157	*∗∗*	2.745	162	*∗∗*	2.807
32–36	1	1401	*∗∗*	3.553	1412	*∗∗*	3.673
22–31	1	1656	*∗∗*	7.664	1669	*∗∗*	8.250
Parity (>1)	1	3	ns	1.048	4	ns	1.056
Year (1988)	1	897	*∗∗*	0.934	760	*∗∗*	0.937
*Nagelkerke R* ^2^		0.326			0.323		

In parentheses the reference category (S: single; BW: birth weight; weeks: duration of gestation). df = degrees of freedom; *p* = statistical significance *∗* < 0.05, *∗∗* < 0.01, ns nonsignificant. OR = relative risk. Wald values taken to the nearest integer. Nagelkerke corrected *R* ^2^ for total and single deliveries.

**Table 4:** Percentage of early neonatal deaths by hours of survival after delivery.

Year	Hours			
	<24	24–47	48–71	72–167
1988	55.710	16.975	10.185	17.130
1989	58.874	15.188	9.898	16.041
1990	60.125	13.570	9.812	16.493
1991	55.906	15.551	9.646	18.898
1992	58.495	15.049	9.223	17.233
1993	55.775	18.310	9.014	16.901
1994	52.962	11.498	14.286	21.254
1995	54.887	13.910	8.647	22.556
2010	38.889	26.389	11.111	23.611
2011	52.041	18.367	9.184	20.408

Data not available for 1996–2009.

**(a):** 

Covariable	df	With multiple			Only single		
		Wald	*p*	OR	Wald	*p*	OR
Multiple (S)	1	37	*∗∗*	1.439	—	—	—
Sex (female)	1	69	*∗∗*	1.312	67	*∗∗*	1.322
BW (2500–4000)	3	3158	*∗∗*		3030	*∗∗*	
>4000	1	47	*∗∗*	1.747	41	*∗∗*	1.692
1500–2499	1	1914	*∗∗*	7.970	1973	*∗∗*	8.429
<1500	1	2852	*∗∗*	34.038	2588	*∗∗*	32.901
Mat. age (<20)	3	292	*∗∗*		266	*∗∗*	
20–29	1	1	ns	0.943	1	ns	0.947
30–39	1	29	*∗∗*	1.389	31	*∗∗*	1.421
40–49	1	117	*∗∗*	0.335	97	*∗∗*	0.358
Weeks (37–41)	3	1552	*∗∗*		1463	*∗∗*	
>41	1	135	*∗∗*	3.177	136	*∗∗*	3.197
32–36	1	637	*∗∗*	3.208	649	*∗∗*	3.304
22–31	1	1437	*∗∗*	11.481	1318	*∗∗*	11.451
Parity (>1)	1	5	*∗*	0.925	3	ns	0.940
Year (1988)	1	590	*∗∗*	0.930	503	*∗∗*	0.930
*Nagelkerke R* ^2^		0.336			0.312		

Left, duration of gestation ≥22 weeks. In parentheses the category of reference (S: single; BW: birth weight; weeks: duration of gestation). df = degrees of freedom; *p* = statistical significance *∗* < 0.05, *∗∗* < 0.01, ns nonsignificant. OR = relative risk. Wald values taken to the nearest integer. Nagelkerke corrected *R* ^2^ for total and single deliveries.

**(b):** 

Covariable	df	With multiple			Only single		
		Wald	*p*	OR	Wald	*p*	OR
Multiple (S)	1	3	ns	1.084	—	—	—
Sex (female)	1	44	*∗*	1.150	46	*∗∗*	1.152
BW (2500–4000)	3	9365	*∗∗*		8903	*∗∗*	
>4000	1	238	*∗∗*	2.058	252	*∗∗*	2.100
1500–2499	1	5854	*∗∗*	8.899	5454	*∗∗*	8.108
<1500	1	8017	*∗∗*	41.130	7701	*∗∗*	36.051
Mat. age (<20)	3	788	*∗∗*		848	*∗∗*	
20–29	1	4	ns	1.076	5	*∗*	1.085
30–39	1	197	*∗∗*	1.790	239	*∗∗*	1.884
40–49	1	105	*∗∗*	0.535	83	*∗∗*	0.577
Weeks (37–41)	3	3472	*∗∗*		3541	*∗∗*	
>41	1	288	*∗∗*	2.911	289	*∗∗*	2.915
32–36	1	1980	*∗∗*	3.412	1938	*∗∗*	3.362
22–31	1	2888	*∗∗*	9.081	3.018	*∗∗*	9.317
Parity (>1)	1	0	ns	1.005	57	*∗∗*	1.184
Year (1988)	1	1384	*∗∗*	0.932	1554	*∗∗*	0.929
*Nagelkerke R* ^2^		0.360			0.350		

Left, duration of gestation ≥22 weeks. In parentheses the category of reference (S: single; BW: birth weight; weeks: duration of gestation). df = degrees of freedom; *p* = statistical significance *∗* < 0.05, *∗∗* < 0.01, ns nonsignificant. OR = relative risk. Wald values taken to the nearest integer. Nagelkerke corrected *R* ^2^ for total and single deliveries.
